# Supplementary material for: Emergence of ferroelectricity in a nonferroelectric monolayer
Source: Nat Commun. 2023 May 13;14:2757. doi: 10.1038/s41467-023-38445-1 (PMC10183010; doi:10.1038/s41467-023-38445-1)
Supplement: Supplementary file 1 — Supplementary Information [file 41467_2023_38445_MOESM1_ESM.pdf]

Supplementary Information for

Emergence of ferroelectricity in a nonferroelectric monolayer

Wenhui Li[1,2†], Xuanlin Zhang[3†], Jia Yang[2,4†], Song Zhou[1,2], Chuangye Song[1,5], Peng Cheng[1,2], Yi-Qi Zhang[1,2], Baojie Feng[1,2], Zhenxing Wang[2,4], Yunhao Lu[3,6*], Kehui Wu[1,2,5*], Lan Chen[1,2,5*]

[1] *Institute of Physics, Chinese Academy of Sciences, Beijing 100190, China*

[2] *School of Physical Sciences, University of Chinese Academy of Sciences, Beijing, 100190, China*

[3] *State Key Laboratory of Silicon Materials, School of Materials Science and Engineering, Zhejiang University, Hangzhou 310027, China*

[4] *National Center for Nanoscience and Technology, Chinese Academy of Sciences, Beijing 100190, China*

[5] *Songshan Lake Materials Laboratory, Dongguan, Guangdong, 523808, China*

[6] *Zhejiang Province Key Laboratory of Quantum Technology and Device, School of Physics, Zhejiang University, Hangzhou 310027, China*

*Emails: lchen@iphy.ac.cn (L.C.); khwu@iphy.ac.cn (K.W.); luyh@zju.edu.cn (Y.L.)

† These authors contributed equally to this work.

Table of Contents

Supplementary Figure 1. The different polytypes of bulk GaSe

Supplementary Figure 2. EDS spectrum of GaSe nanoflake

Supplementary Figure 3. The low-magnification STEM images of GaSe nanoflake

Supplementary Figure 4. Experimental setup for SHG measurement schematic

Supplementary Figure 5. Ferroelectric polarization switching by PFM for monolayer γ-GaSe

Supplementary Figure 6. Charge transfer process in the ferroelectric polarization reversal

Supplementary Figure 7. The relative energy change of the ferroelectric phase transition process

Supplementary Figure 8. Theoretically calculated polarization reversal barrier of monolayer GaSe

Supplementary Figure 9. Variation of local potential along the out-of-plane direction for few-layer GaSe

Supplementary Figure 10. Charge density difference between the distorted Q-phase and the remaining layers

Supplementary Figure 11. AFM images with height profile of γ-GaSe

Supplementary Figure 12. The sweeping rate-dependent electrical tests

Supplementary Figure 13. The resultant band alignments of the GaSe FET device

Supplementary Figure 14. The repeatability of ferroelectric resistance switching

Supplementary Figure 15. The retention performance of HRS

Supplementary Figure 16. Optical absorption spectrum and Photoluminescence (PL) of GaSe nanoflake

Table S1. Charge transfer and OOP polarization in few-layer GaSe

Supplementary Note 1. The reasons why ferroelectric domains can’t flip 180° phase

Supplementary Note 2. The oxidation process of GaSe

Supplementary Figure 1

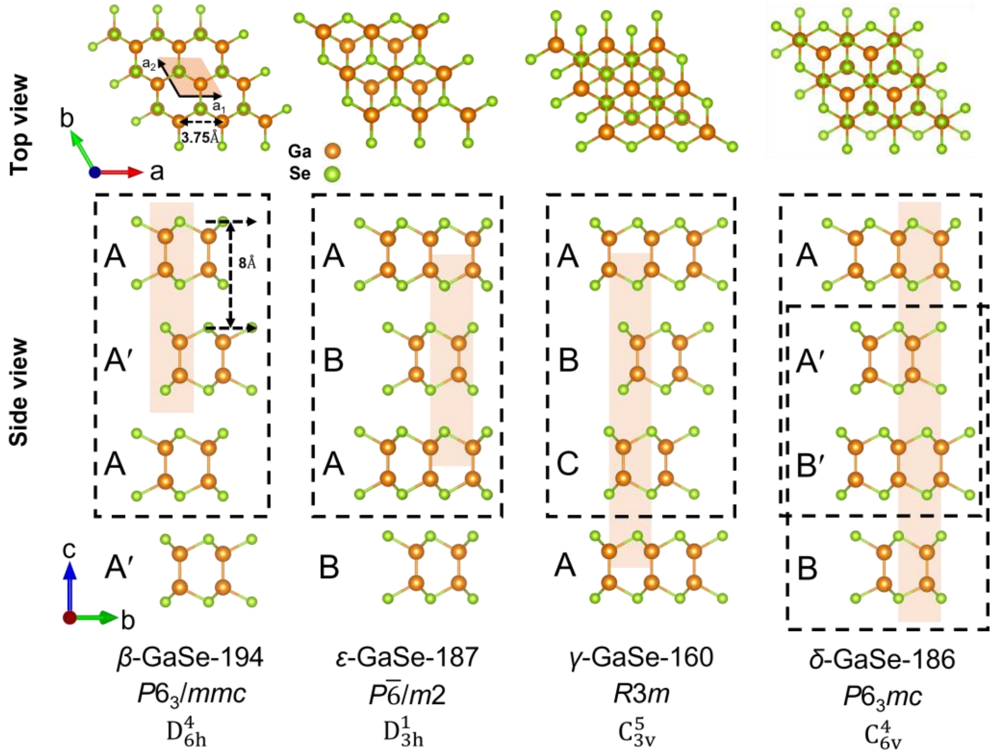

Supplementary Figure 1. The different polytypes of bulk GaSe.

Generally speaking, bulk GaSe mainly has four different polytypes, designated as β(D_{6h}^4), ε(D_{3h}^1), γ(C_{3v}^5), and δ(C_{6v}^4). Different GaSe polytypes have the same primitive unit cells but different interlayer stacking sequences.

Supplementary Figure 2

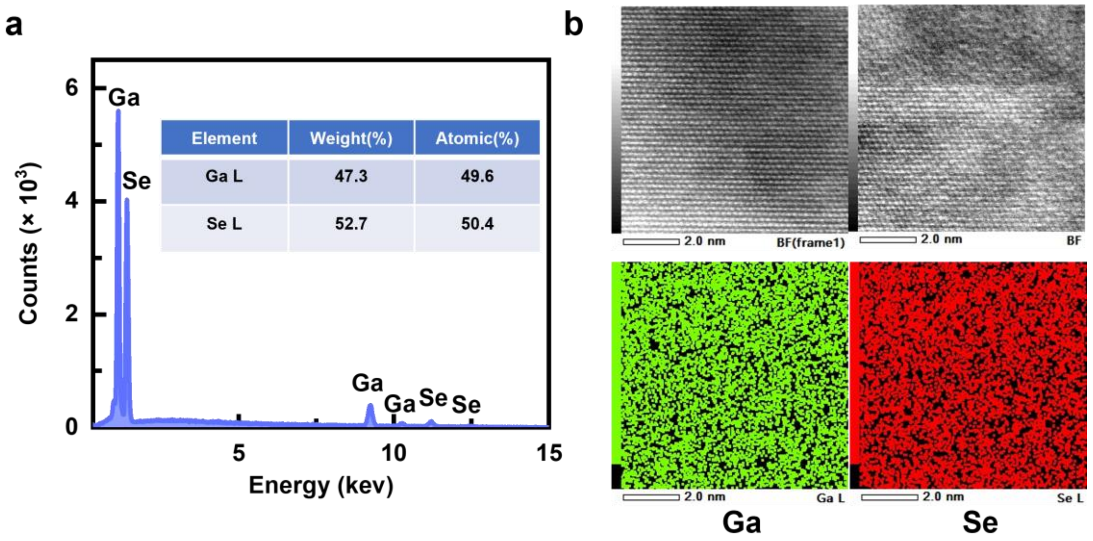

Supplementary Figure 2. EDS spectrum of GaSe nanoflake. (a) The EDS spectrum of GaSe nanoflake. **(b)** The STEM-EDS mapping of Ga and Se elementals.

The EDS spectrum shows only Gallium and selenium elements without impurity peaks were detected, confirming high purity of the GaSe nanoflake (Supplementary Fig.

2a). The atomic ratio of Ga:Se chemical composition is about 1:1, which is very approach to the nominal component of GaSe. The STEM-EDS mapping of Ga and Se elementals demonstrate the uniform element distribution of GaSe (Supplementary Fig. 2b).

Supplementary Figure 3

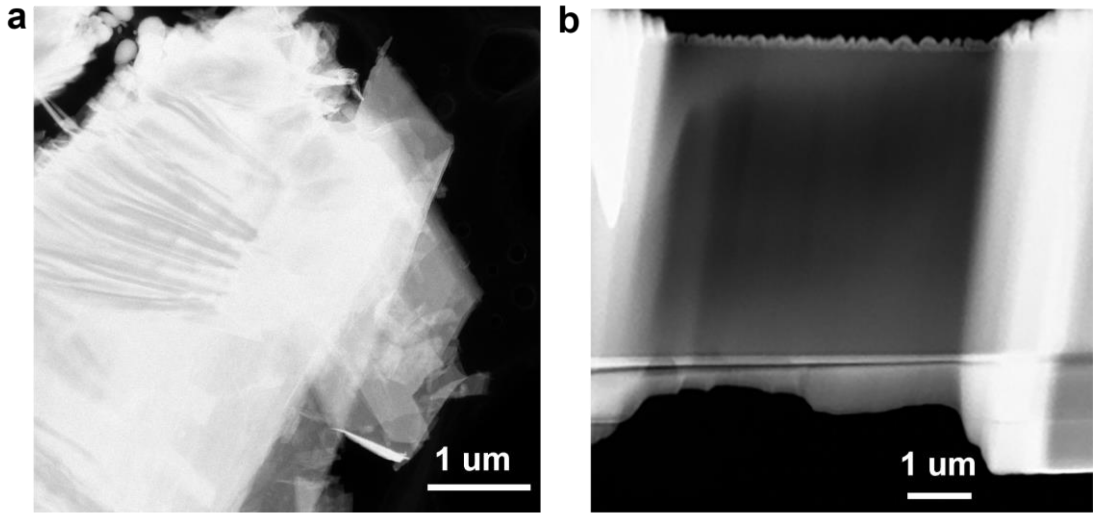

Supplementary Figure 3. The low-magnification STEM image of GaSe nanoflake. (a) The planar-view STEM image of GaSe nanoflake along the [001] zone axis. **(b)** The cross-section STEM image of GaSe nanoflake along the [100] zone axis.

Supplementary Figure 4

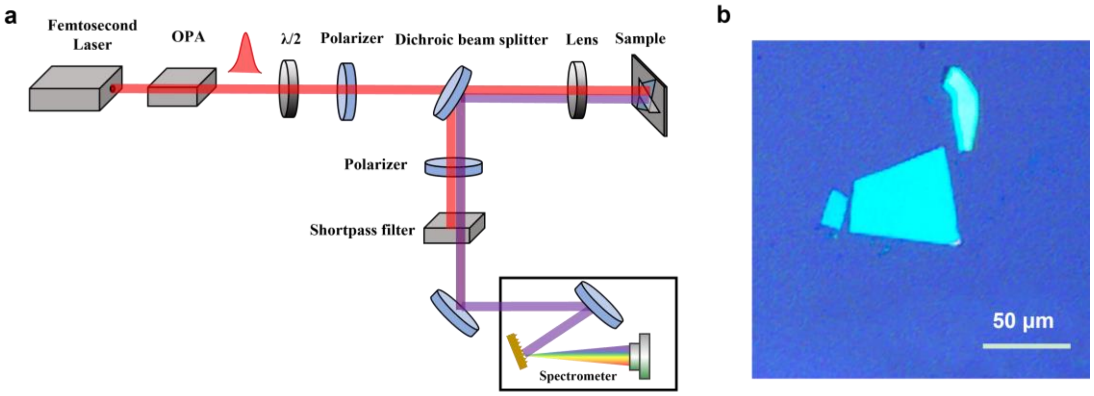

Supplementary Figure 4. Experimental setup for SHG measurement schematic. (a) Experimental setup for SHG measurement schematic. **(b)** The optical microscopy image of GaSe nanoflake for SHG signal detection.

The excitation ultrafast light at 800 nm is converted into polarized light through a polarizer, and then converted into polarized light in a specific direction by rotating a half-wave plate (Supplementary Fig. 4a). The excitation light is then focused on the sample through the objective lens (100×, N.A.=95) with a spot size about 2 um. The frequency-doubled SHG signal at 400 nm generated by the sample in the backscattering configuration and then separated by a dichroic beam splitter. The SHG signal was collected by placing another polarizer in parallel with the excitation light. The

excitation light is filtered out with a short-pass filter before the SHG signal reaches the spectrometer.

Supplementary Figure 5

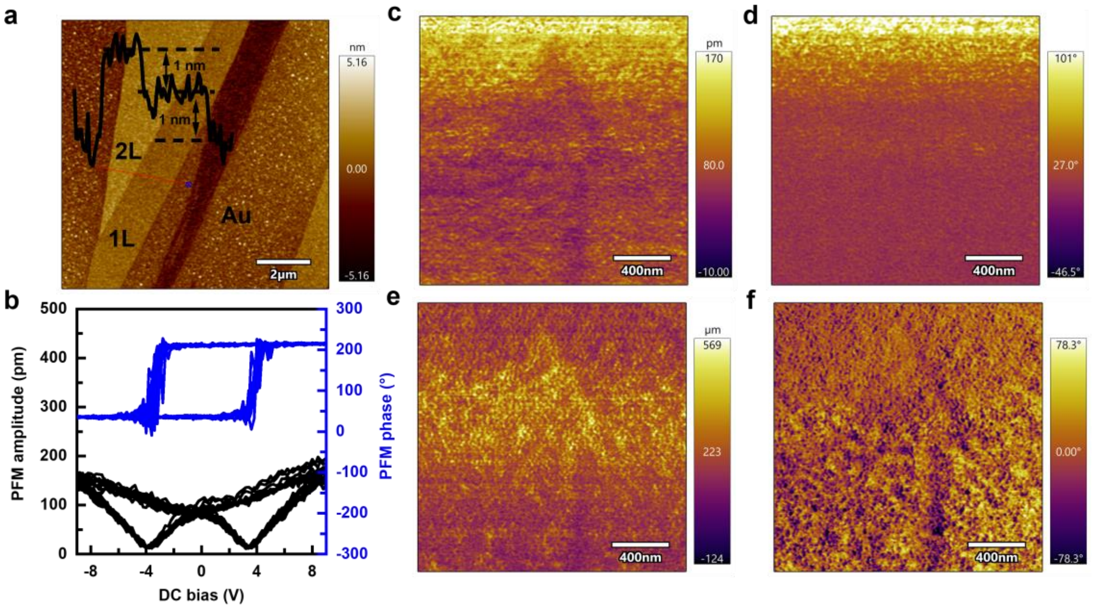

Supplementary Figure 5. Ferroelectric polarization switching by PFM for monolayer γ-GaSe. (a) AFM topography of monolayer γ-GaSe nanoflake. **(b)** The butterfly-shaped amplitude-voltage loops and phase hysteresis loops performed in monolayer γ-GaSe at room temperature. **(c, d)** The PFM OOP amplitude and phase images of monolayer γ-GaSe after writing arrow symbol pattern with opposite DC bias (+10 V and -10V), respectively. **(e, f)** The PFM IP amplitude and phase images of monolayer γ-GaSe after writing arrow symbol pattern with opposite DC bias (+10 V and -10V), respectively.

Supplementary Figure 6

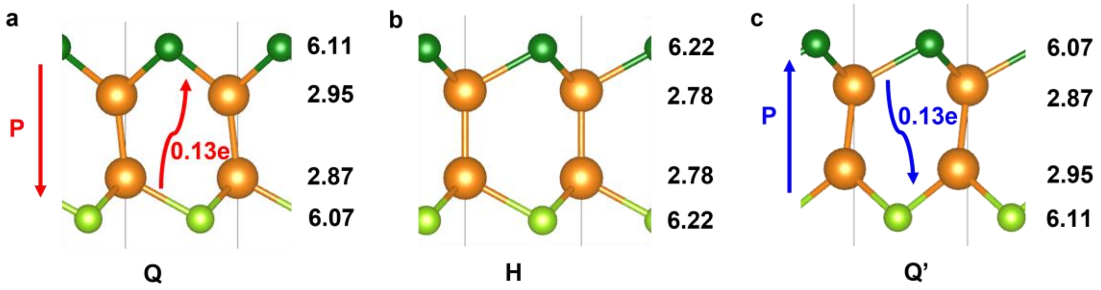

Supplementary Figure 6. Charge transfer process in the ferroelectric polarization reversal. The Mulliken population on each atom in **(a)** Q, **(b)** H and **(c)** Q' phase. The curved arrows indicate the direction of charge transfer between GaSe sublayers. The straight arrows indicate the direction of OOP polarization.

Supplementary Figure 7

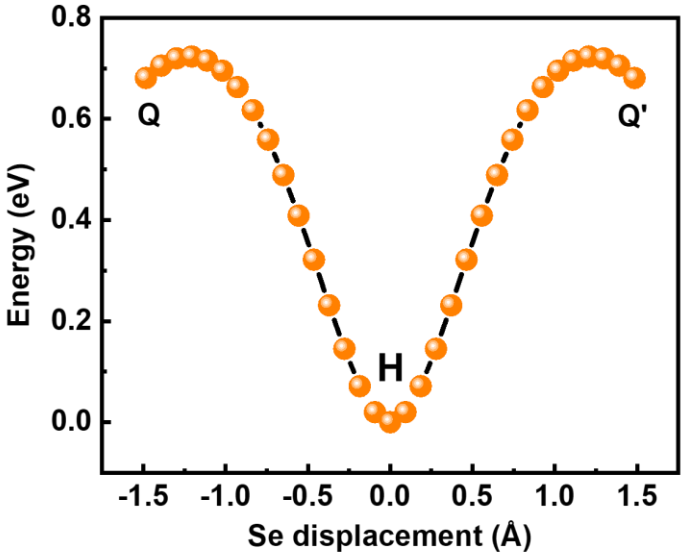

Supplementary Figure 7. The relative energy change of the ferroelectric phase transition process. The relative energy of structures between the Q/Q' and the H phase. At each point, in-plane coordinates of atoms are fixed, and the out-of-plane coordinates are fully relaxed.

Supplementary Figure 8

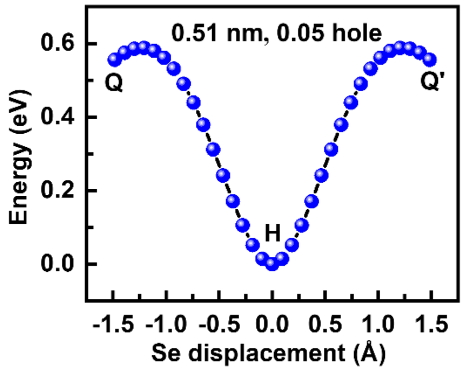

Supplementary Figure 8. Theoretically calculated polarization reversal barrier of monolayer GaSe. The reversal barrier under p-type doped GaSe condition (0.05hole/u.c), where the thickness of monolayer GaSe is taken as 0.51 nm.

Supplementary Figure 9

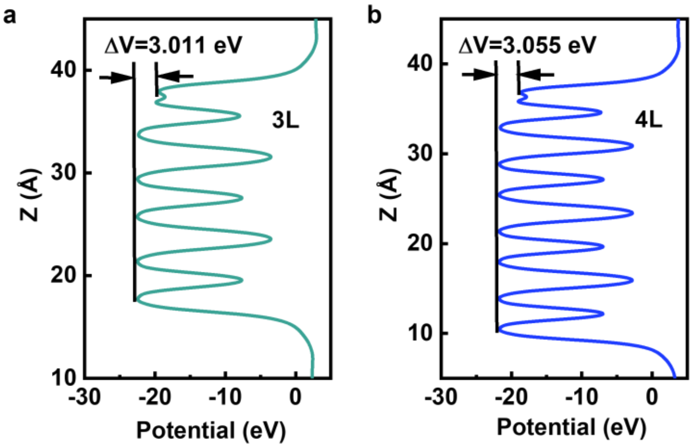

Supplementary Figure 9. Variation of local potential along the out-of-plane direction for few-layer GaSe. The local potential variations along the Z-direction for the 3L and 4L Q-phase GaSe.

Supplementary Figure 10

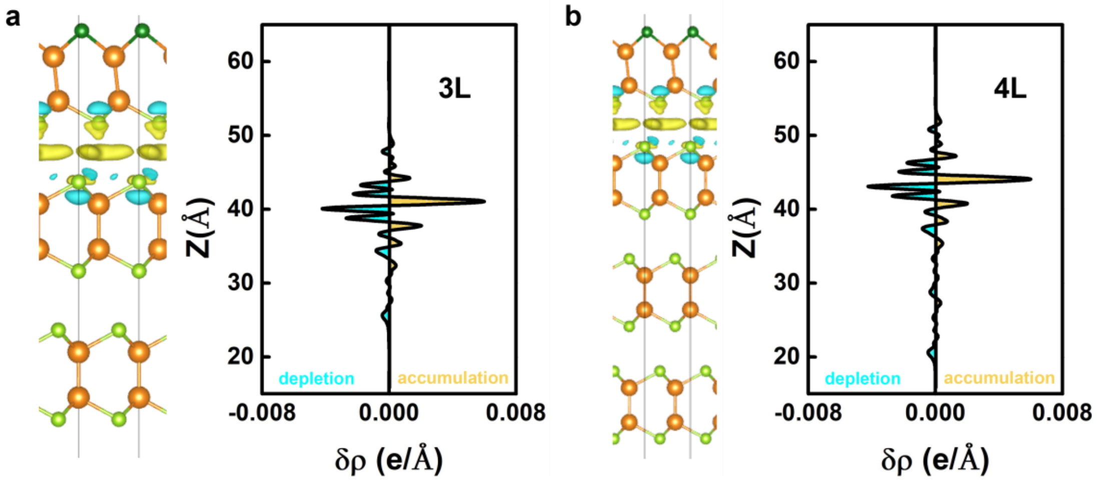

Supplementary Figure 10. Charge density difference between the distorted Q-phase and the remaining layers. The differential charge density between the distorted Q-phase and the rest of layers in real space (isosurface 0.0001 e/Bohr^3, left panel) and corresponding planar averaged differential charge density (right panel) in **(a)** 3L and **(b)** 4L GaSe films. The light blue and yellow colors represent electron accumulation and depletion regions, respectively.

Table S1. Charge transfer and OOP polarization in few-layer GaSe. The charge transfer calculated by integrating the differential charge between the topmost distorted Q-phase and the rest of layers and the OOP polarization in 2L, 3L and 4L Q-phase GaSe.

Layers	Charge transfer (e)	Polarization (pC/m)
2L	0.0005	5.47
3L	0.0146	6.19
4L	0.0181	7.17

Supplementary Figure 11

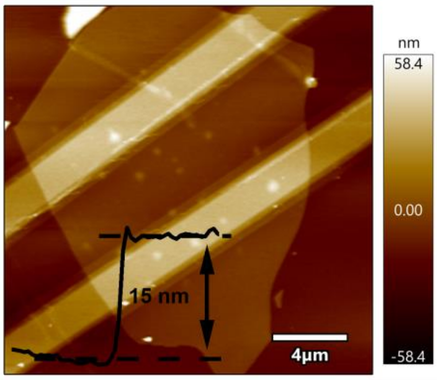

Supplementary Figure 11. AFM images with height profile of γ-GaSe. The height of γ-GaSe nanoflake for GaSe FET device is 15 nm.

Supplementary Figure 12

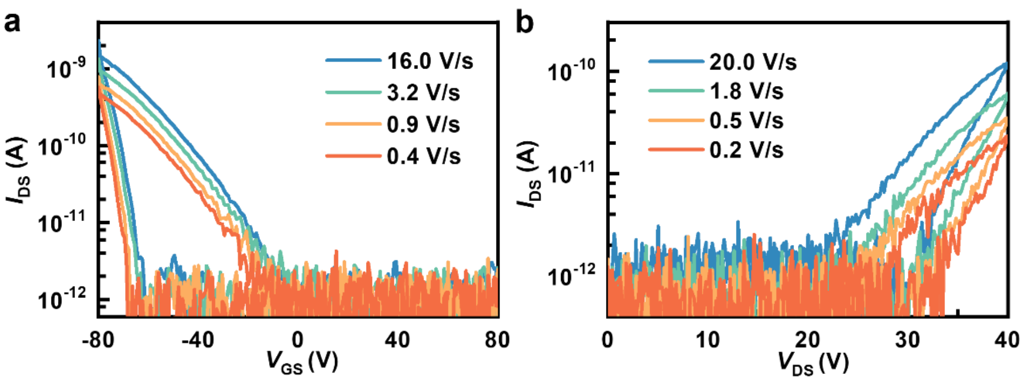

Supplementary Figure 12. The sweeping rate-dependent electrical tests. The sweeping rate-dependent **(a)** transfer at V_{DS} =10 V and **(b)** output curves at V_{GS} = 0 V.

We performed sweeping rate-dependent electrical tests shown in Supplementary Fig. 12. Due to the charge trapping effect, the hysteresis loop window typically becomes larger when the voltage sweep is slower[1,2]. However, it can be seen from our test results that for both the transfer and output curves, the hysteresis loops window does not shift obviously during the voltage sweeping process, thus ruling out the effect of trap states.

Supplementary Figure 13

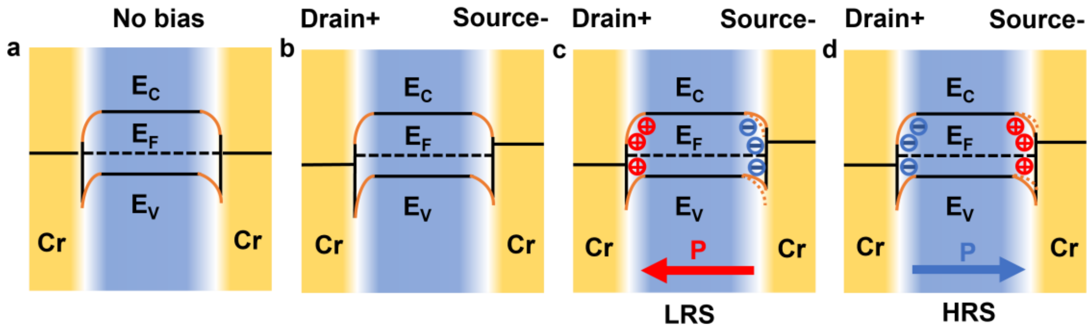

Supplementary Figure 13. The resultant band alignments of the GaSe FET device. (a) Without bias, the Fermi levels of the electrode and GaSe are aligned. **(b)** Band bending under positive drain bias. **(c, d)** The negative (positive) poling induces negative (positive) polarization charges accumulate at the source, thereby lowering (raising) the Schottky barrier and resistance, switching the device to the LRS (HRS) state, respectively.

Generally speaking, the Cr metal electrode has a relatively lower metal work function(4.5eV) than the Au electrode (5.1 eV). Therefore, the memristor FeFET device can be realized by using the Schottky contact effect formed at the metal/semiconductor interface[3,4]. In addition, the schematic of the band alignments at different bias with polarization switching of the lateral device.The bandgap alignments of ferroelectric semiconductors can be changed as the polarization direction switched. The ferroelectric polarization charges will cause the asymmetric Schottky barrier. Moreover, the asymmetric Schottky barriers height will be modulated by ferroelectric polarization switching, then resulting in HRS and LRS. Firstly, when the metal Cr electrode contact to GaSe semiconductor without bias, the Fermi levels of the metal and semiconductor will be pulled at the same level (Supplementary Fig. 13a). When the negative bias poling is applied to the source and drain, the negative polarized charges will accumulate at the source, which results in Schottky barriers and resistance decreases, and then the device in LRS (Supplementary Fig. 13c). Conversely, when the positive bias poling is applied to the source and drain, the positive polarized charges will accumulate at the source, resulting in Schottky barriers and resistance increases, and then the device in HRS (Supplementary Fig. 13d). It can be seen from the transfer characteristic curve I_{DS}-V_{GS} that the GaSe nanoflake exhibits typical p-type semiconductor behavior (Fig. 5d), which the channel current increases with increasing gate voltage under the gate voltage range.

Supplementary Figure 14

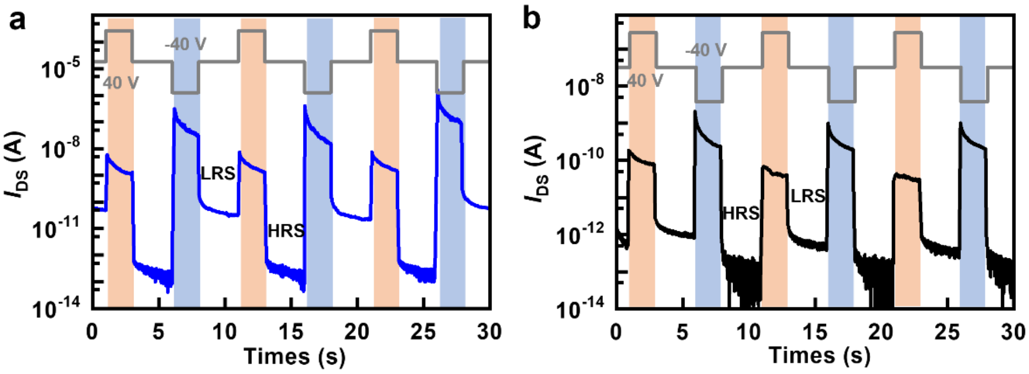

Supplementary Figure 14. The repeatability of ferroelectric resistance switching. (a) The resistance switching by applying the channel bias. **(b)** The resistance switching by applying the gate voltage.

We repeatedly pole the IP resistance switching by applying a periodic pulse channel bias of ±40 V, then read by 10 V. The resistance switching exhibits a high LRS/HRS ratio of 10^3 by applying channel bias (Supplementary Fig. 14a). The lateral GaSe ferroelectric memristor based on IP polarization exhibits gate tunability by applying periodic pulse gate bias, and the IP resistance switching exhibits a considerable LRS/HRS ratio of 10^2 by applying the gate voltage (Supplementary Fig. 14b).

Supplementary Figure 15

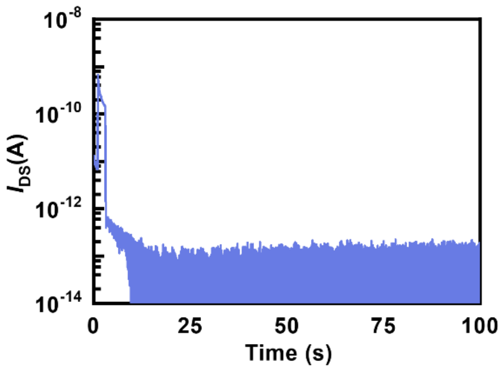

Supplementary Figure 15. The retention performance of high resistance state (HRS) for over 100 s. A drain voltage of 10 V has been applied ahead for 2 s to set the HRS.

Supplementary Figure 16

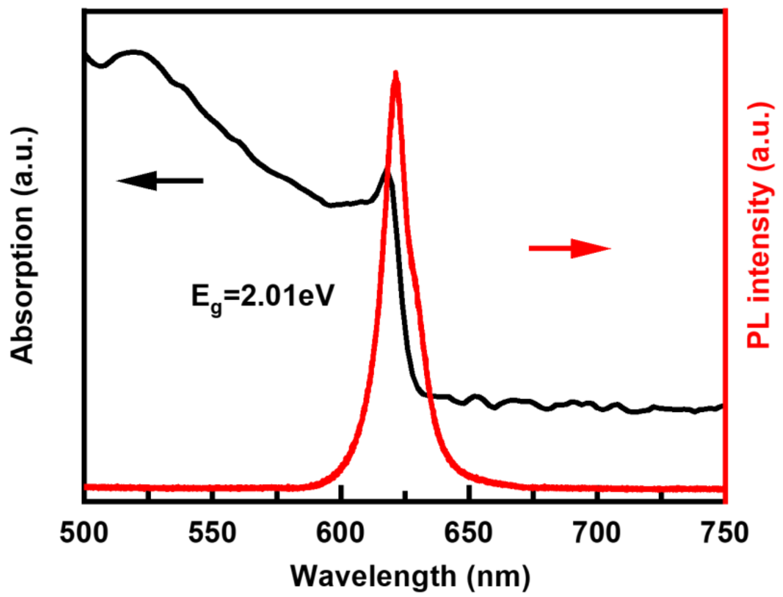

Supplementary Figure 16. Optical absorption spectrum and PL of GaSe nanoflake.

Optical absorption spectrum of GaSe nanoflake showing direct band gap of 2.01eV at room temperature. PL spectrum of GaSe excited with 325 nm laser light, showing that a significant excitation peak observed slightly below 2.01EV due to the influence of exciton binding energy.

Supplementary Note 1. The reasons why ferroelectric domains can't flip 180° phase.

The distinct 180° phase switching can be observed in the switching spectroscopy. Nevertheless, the ferroelectric domains can't attain the 180° phase switching in the PFM "read and write" operations. We think there are several possible reasons as follows: First, the local SS-PFM was collected in dual alternating current (AC) resonance tracking mode by superimposing an AC signal on a series of DC triangular sawtooth waveform voltages, so the actual maximum voltage peak is larger than the DC voltage. The maximum range of PFM DC voltage is ±10 V, the SS-PFM polarization loops reversal could be measured point by point in this range. However, the voltage is not enough to reverse polarization of PFM amplitude and phase images in the "read and write" operations. Second, for the SS-PFM test, the voltage is applied for a relatively long, and the signal collected is nearly transient after application of the DC voltage[5]. In contrast, for the "read and write" operations, the PFM tip swept away quickly causing the dwell time at each point is relatively short. The PFM amplitude and phase images are read in relaxation state after the DC bias voltage is tuned off. The polarized electric field may only penetrate the surface of the sample, but it is too late to completely penetrate the interior of the sample, which leads to the polarization of ferroelectric domains can't be fully reversed. Third, if the polarization direction of the lowest energy is not vertical but canted, the OOP remanent polarization will decay after the DC bias voltage is turned off. Since PFM measurement is sensitive to the OOP polarization component, resulting in the OOP polarization signal is weaker than the IP signal. In

conclusion, it's not easy to observe the ferroelectric domains with 180° phase fully reversed by PFM "read and write" operations in GaSe nanoflakes.

Supplementary Note 2. The oxidation process of GaSe

The oxidation process of GaSe is influenced by multiple factors, such as oxygen, humidity, and photo-induced[6]. We prepared monolayer GaSe on HOPG substrate by MBE method (Supplementary Fig. 17a), and investigated the oxidation process by XPS. Supplementary Fig. 17(b, c) shows the XPS spectra of monolayer GaSe exposure to air with evolution in time. After 25 h exposure to air, the Se $3d^{2/3}$ and Se $3d^{2/5}$ doublet peaks began to appear no longer obviously separated, and gradually merged into one peak. In addition, the positions of both Se 3d and Ga 3d peak shift towards lower binding energy, indicating the change of the chemical valence state of Ga and Se, and the appearance of oxidation products, such as Ga_2O_3 and a-Se[7]. However, our PFM experiments on GaSe monolayer is performed on the sample exposure to air within one hour. Therefore, we believe the sample of GaSe monolayer does not degrade during PFM measurements.

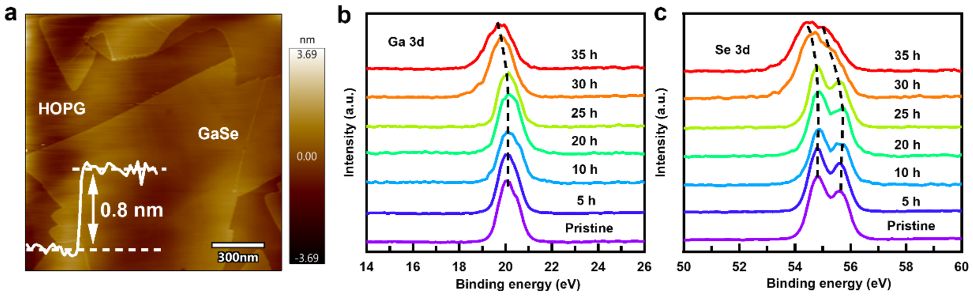

Supplementary Figure 17. Evolution of XPS spectra during GaSe oxidation. (a) Morphology of monolayer GaSe on HOPG substrate. **(b, c)** Evolution in time of XPS spectra of Ga 3d and Se 3d core levels during GaSe oxidation.

We also investigated exfoliated GaSe sample with thickness about 3.6 nm (Supplementary Fig. 18(a, b)) by Raman spectroscopy characterization. As shown in the Supplementary Fig. 18c, The Raman spectra of few-layer GaSe show four Raman characteristic vibrational modes at 19.4 cm^{-1} (E_{2g}^2), 134.8 cm^{-1} (A_{1g}^1), 214 cm^{-1} (E_{2g}^1), 309 cm^{-1} (A_{1g}^2), while missing three Raman phonon modes appeared in thicker samples at 59.1 cm^{-1} (E_{1g}^1), 235 cm^{-1} (A_2''(TO)), 247.5 cm^{-1} (A_2''(LO)). Beyond that associated with GaSe, additional Raman modes were observed at 202 and 250 cm^{-1}. The broad peak near 250 cm^{-1} with a shoulder on the low-wavenumber side after 48 h exposure to the air should be the Raman feature of a-Se[8], while the Raman peak at 202 cm^{-1} should be identified as the characteristic vibrational mode of Ga_2O_3[9].At room temperature, the main products of GaSe oxidation are Se and Ga_2O_3[10].Moreover, with a slight increase in excitation light power, GaSe oxidizes rapidly in a few minutes, indicating that it is highly susceptible to photoinduced oxidation, as shown in Supplementary Fig. 18d. Therefore, prolonged light tests or strong light tests can lead to GaSe oxidation quickly, and the surface after photo-induced oxidation lead to that the particle cluster

morphology occurs, as shown in the area marked by small circles in Supplementary Fig. 18(a, b).

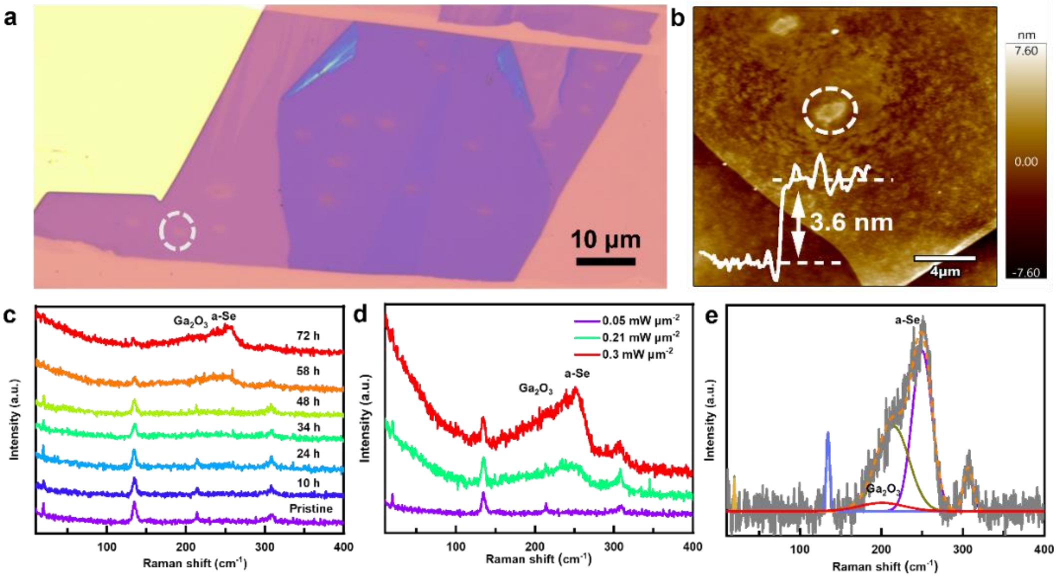

Supplementary Figure 18. **Evolution of Raman spectra during GaSe oxidation. (a, b)** The Optical micrograph and AFM morphology of exfoliated GaSe sample with a thickness of about 3.6 nm, respectively. The oxidized area after Raman light illumination is marked by small circles. **(c)** Evolution in time of Raman spectra of GaSe, tested with 532 nm laser and power density of 0.05 mW μm^{-2}. **(d)** Raman spectra of GaSe at power density of 0.05, 0.21, and 0.3 mW μm^{-2}, respectively. **(e)** Peak fitting of Raman spectra of oxidized GaSe. The short dash line represents the total fit of the data, additional peaks appear at 202 and 250 cm^{-1} identified as Ga_2O_3 and a-Se, respectively.

The optical techniques for characterizing the GaSe oxidation such as PL, XPS and Raman, can accelerate the oxidation by photo-inducement[6]. However, our PFM experiments on GaSe sample are performed in a dry environment, and no laser introduced, in which the sample cannot be much degraded within 25 hours. Therefore, our experimental measurements should reflect the pristine properties of GaSe.

Supplementary references

1. Meng, P. et al. Sliding induced multiple polarization states in two-dimensional ferroelectrics. *Nat Commun*. **13**, 7696 (2022).

2. Alam. MNK. et al. On the Characterization and Separation of Trapping and Ferroelectric Behavior in HfZrO FET. *IEEE. J. Electron. Devi.* **7**, 855-862 (2019).

3. Chen, A. et al. Couplings of polarization with interfacial deep trap and Schottky interface controlled ferroelectric memristive switching. *Adv. Funct. Mater.* **30(43)**, 2000664 (2020).

4. Si, M. et al. Asymmetric metal/α-In_2Se_3/Si crossbar ferroelectric semiconductor junction. *ACS Nano* **15 (3)**, 5689−5695 (2021).

5. Vasudevan, R. K., Balke, N., Maksymovych, P., Jesse, S. & Kalinin, S. V. Ferroelectric or non-ferroelectric: Why so many materials exhibit “ferroelectricity” on the nanoscale. *Appl. Phys. Rev.* **4**, 021302 (2017).

6. Beechem, T. E. et al. Oxidation of ultrathin GaSe. *Appl. Phys. Lett.* **17(107)**, 173103

(2015).
7. Rahaman, M. et al. GaSe oxidation in air: from bulk to monolayers. *Semicond. Sci. Tech.* **32(10)**, 105004 (2017).
8. Lukács, R. et al. On photoinduced volume change in amorphous selenium: Quantum chemical calculation and Raman spectroscopy. *J. Appl. Phys.* **107(7)**, 073517(2010).
9. Onuma, T. et al. Polarized Raman spectra in β-Ga_2O_3 single crystals. *J. Cryst. Growth.* **401**, 330-333 (2014).
10. Andres-Penares, D. et al. Quantum size confinement in gallium selenide nanosheets: band gap tunability versus stability limitation. *Nanotechnology.* **28(17)**, 175701(2017).
